# Three dimensional and microphysiological bone marrow models detect in vivo positive compounds

**DOI:** 10.1038/s41598-021-01400-5

**Published:** 2021-11-09

**Authors:** Rhiannon David, Sarah Gee, Kainat Khan, Amy Wilson, Ann Doherty

**Affiliations:** Clinical Pharmacology and Safety Sciences, R&D, AstraZeneca, Cambridge, UK

**Keywords:** Lab-on-a-chip, Toxicology, DNA damage and repair

## Abstract

Micronucleus (MN) assessment is a valuable tool in safety assessment. However, several compounds are positive in the in vivo bone marrow (BM) MN assay but negative in vitro, reflecting that BM complexity is not recapitulated in vitro. Importantly, these compounds are not genotoxic; rather, drug-driven pharmacological-effects on the BM increase MN, however, without mechanistic understanding, in vivo positives stop drug-progression. Thus, physiologically-relevant BM models are required to bridge the gap between in vitro and in vivo. The current study aimed to investigate the utility of two human 3D BM models (fluidic and static) for MN assessment. MN induction following treatment with etoposide and Poly-ADP Ribose Polymerase inhibitor (PARPi) and prednisolone (negative in vitro, positive in vivo) was determined in 2D L5178Y and human BM cells, and the 3D BM models. Etoposide (0–0.070 µM) and PARPi (0–150 µM) induced MN in both 3D BM models indicating their utility for genotoxicity testing. Interestingly, PARPi treatment induced a MN trend in 3D more comparable to in vivo. Importantly, prednisolone (0–1.7 mM) induced MN in both 3D BM models, suggesting recapitulation of the in vivo microenvironment. These models could provide a valuable tool to follow up, and eventually predict, suspected pharmacological mechanisms, thereby reducing animal studies.

## Introduction

Genetic toxicity testing in drug development evaluates the potential for a compound to cause genetic damage in humans, and in industry the standard test battery (bacterial mutagenesis, in vitro cytogenetics, and in vivo genotoxicity) is well established. Micronucleus (MN) assessment, part of the battery, is typically performed in both in vitro 2-dimensional (2D) cell culture and in vivo rodent assays. MN assessment is a valuable tool due to its capacity to detect both anuegenic and clastogenic chromosome damage^1^, and plays an important role predicting potential risks in human.

Despite this, a number of compounds are negative in vitro but positive in vivo^2^, such as the glucocorticoid receptor agonists, prednisolone and dexamethasone. These are not truly genotoxic or carcinogenic, as evidenced by the lack of carcinogenic risk in humans following decades of clinical use^3,4^, but are the result of pharmacological effects on the BM leading to an increase in MN in vivo^3^. This is further supported by observations where post-dexamethasone treatment there was increase in erythropoiesis in haematopoietic cells from mouse foetal livers in vitro^5^, and in a mouse model with Diamond-Blackfan Anaemia (DBA), which have depleted erythroid progenitors, glucocorticoid treatment reversed the effect leading to an increase in erythrocytes^6^. Other examples of increased BM cell proliferation leading to increased MN frequency include studies examining the use of the growth factor erythropoietin, which increases erythropoiesis and MN frequency^7^. This discordance between in vitro and in vivo, particularly for the compounds that are not intrinsically genotoxic, has been attributed to changes in body temperature, resulting in an increase in micronucleated IEs, in both rodents with hyperthermia^8–10^ and hypothermia^11,12^. Although compounds that induce MN via these mechanisms are not truly genotoxic or carcinogenic, an in vivo positive result stops their progression into the clinic. In order to predict for pharmacological mechanisms in the BM, and de-risk an in vivo positive when there is an in vitro negative, there is a need for a model that mimics the complex BM microenvironment, which consists of a heterogeneous cell population representing different lineages and stages of differentiation, since the standard in vitro MN assay typically uses a single cell type, either the L5178Y or TK6 cell lines, or primary human lymphocytes. In recent years there has been an increase in the development of three-dimensional (3D) cell culture systems, which aim to recapitulate tissue function by creating complex tissue/organ microenvironments. These include organs-on-a-chip, scaffold-based systems, spheroids and organoids^13,14^. Investigation into the use of these models for genetic toxicology has demonstrated some success: a 3D skin model for a skin comet assay assessing DNA damage^15^ and a MN assay^16^, while more recently, 3D liver spheroids have been investigated for MN detection^17^. However, the role of a 3D BM model for genotoxicity testing remains to be explored. MN assessment in a 3D BM model would help bridge the gap between current in vitro and in vivo studies and could be used to capture in vivo-only MN positives induced by non-genotoxic compounds. To address this, we have developed a fluidic and static 3D BM model using a hard ceramic scaffold representative of the human BM, which were adapted from the model developed by Sieber et al.^18^. They comprise CD34 + haematopoietic stem cells (HSPCs) seeded onto a mesenchymal stem cell (MSC)-coated hard ceramic scaffold co-cultured in either a microfluidics system (TissUse HUMIMIC Chip2) or in static culture (24 well plate).

The aim of the current study was to test the application of these two 3D BM models to genotoxicity testing, investigated using two genotoxic compounds that act by different mechanisms, etoposide and a Poly-ADP Ribose Polymerase inhibitor (PARPi) that has not been taken forward into the clinic. Etoposide inhibits Topoisomerase II, an enzyme involved in DNA transcription, replication and remodelling^19^, while the PARPi interferes with base excision repair (BER), leading to increased DNA damage and error-prone repair in homologous recombination (HR)-deficient cells by non-homologous end joining (NHEJ) (reviewed in Sistigu et al.^20^). Furthermore, the ability of these 3D models to detect in vivo pharmacological positives, tested using the steroid prednisolone was also investigated.

## Results

### MN are detected in both 3D bone marrow models following treatment

**Figure 1:**
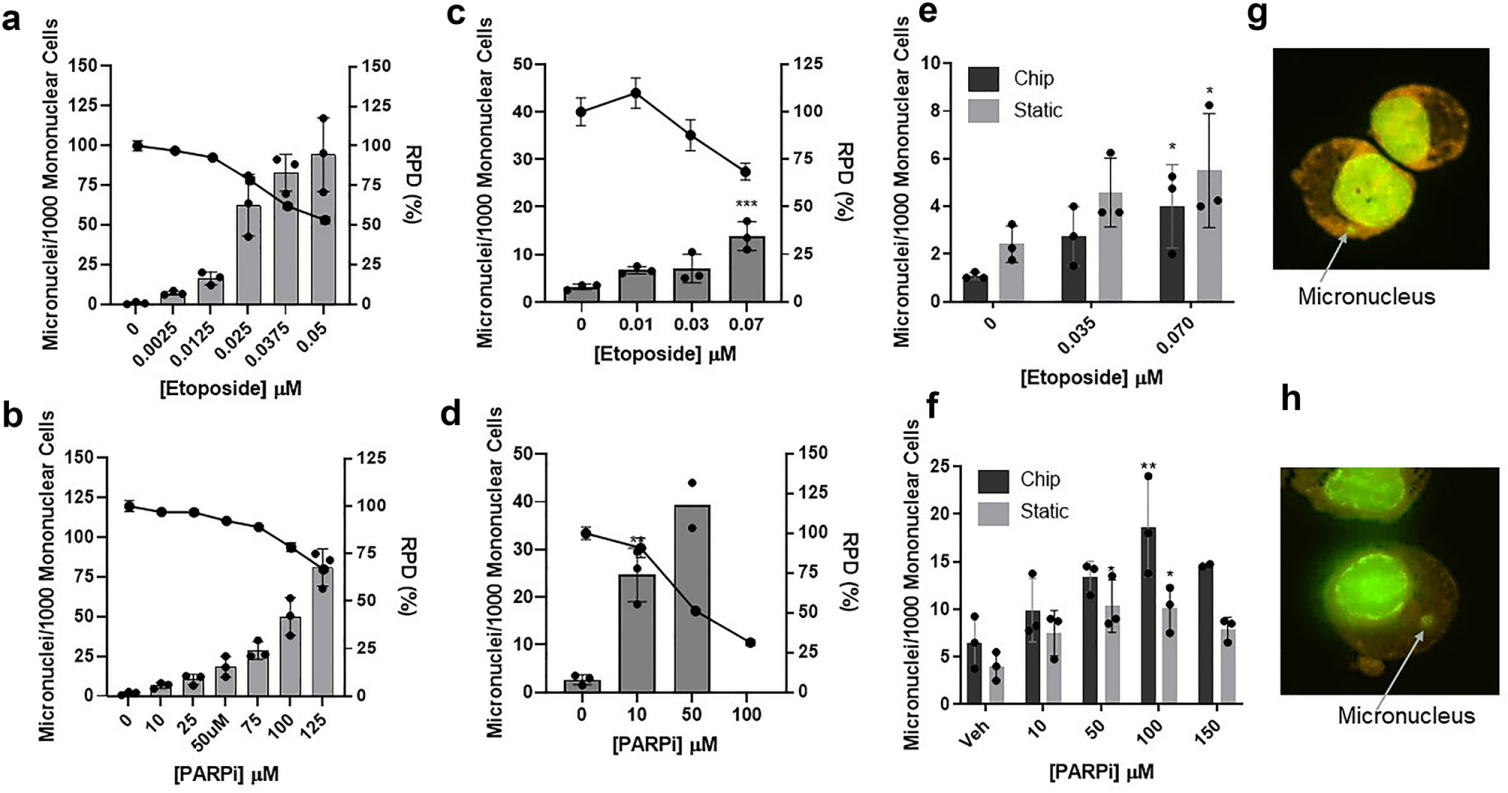
Micronuclei are detected in both 3D bone marrow models following treatment with etoposide or a PARP inhibitor. Micronuclei (MN) induction with concurrent cell viability in L5178Y cells following treatment with etoposide (**a**) or PARPi (**b**), in 2D BM cells following treatment with etoposide (**c**) or PARPi (**d**), and in the 3D chip and static models following treatment with etoposide (**e**) or PARPi (**f**). Representative MN images (×40) from the chip (**g**) and static (**h**) models. Data are presented as mean ± SD, n = 3. Bars represent MN/1000 cells; lines represent relative population doubling (RPD), a measure of cytotoxicity in the L5178Y assay, or % cell viability (ATP measurement) in the 2D BM assay; images of MN were stained with acridine orange, which stains double stranded DNA green (nucleus) and RNA red/orange (cytoplasm), arrows indicate a MN and are representative of MN scored; n = 3 (n = 2 for 50 µM PARPi in 2D, 150 µM PARPi in chip). Significance compared to vehicle control (one way ANOVA with Dunnett’s post-test; *p < 0.05; **p < 0.01; ***p < 0.001; ****p < 0.0001).

Etoposide and a PARPi were used in the standard in vitro MN assay in L5178Y cells to determine their genotoxic and cytotoxic effects. As expected, treatment with both compounds induced a dose-dependent increase in MN and Relative Population Doubling (RPD) (Fig. 1a, b). Statistically significant increases in MN were observed for 0.025, 0.0375, 0.05 and 0.065 µM etoposide and 75, 100 and 125 µM PARPi compared to the vehicle control (values were also greater than the laboratory historical vehicle-treated control range for MN/1000 cells (2.85 MN ± 2.26 SD)). MN inductions were all observed at between 0 and 55.5% Relative Population Doubling (RPD), and 55 ± 5% is the cytotoxicity limit recommended by the OECD guideline for this assay^21^. A statistically significant, dose-dependent increase in MN was also observed in the 2D BM cell in vitro MN assay following treatment with 0.07 µM etoposide, and 10 and 50 µM PARPi (Fig. 1c, d). Cytotoxicity induced at these concentrations, measured by ATP concentration, was below the 55 ± 5% cytotoxicity limit. No MN data were generated for 100 µM PARPi as cytotoxicity was greater than this limit.

To investigate the applicability of the 3D chip and static models to genotoxicity testing, cells were scored for MN following treatment with etoposide and the PARPi. A dose-dependent increase in MN was detected for both drugs in both models following treatment (Fig. 1e, f). Statistically significant increases in MN were observed for 0.07 µM etoposide in both models, while treatment with PARPi induced statistically significant increases in MN for 50 µM and 100 µM in the static model, and 100 µM only in the chip model, all compared to the vehicle control. Representative images depicting MN observed in the chip following treatment with etoposide and the static in response to the PARPi are shown in Fig. 1g, h respectively.

### Viability is greater than 50% in the 3D bone marrow models following treatment

**Figure 2:**
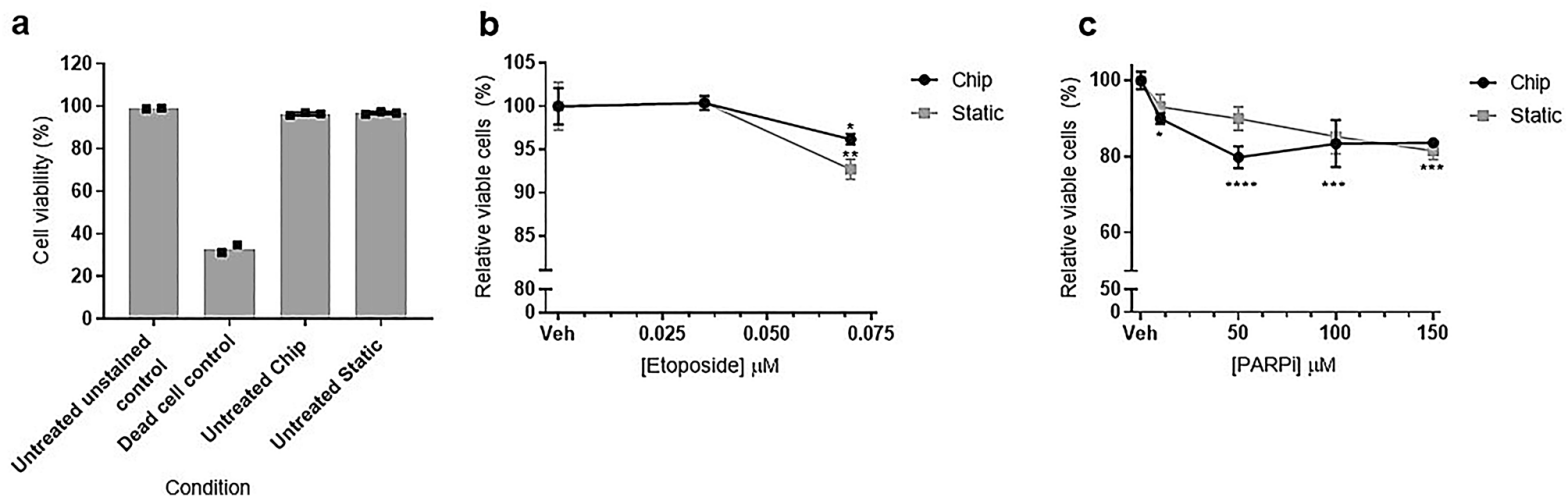
Cell viability is maintained during 3D BM culture, and is > 50% following Etoposide and PARPi treatment. Cytotoxicity was determined by flow cytometry using 7-AAD^+^ viability staining to detect dead cells (%). Cell viability in (**a**) untreated cells on day 8 (n = 3 for chip and static, n = 2 for 7-AAD control (freeze-killed cells) and unstained control (untreated cells)) of culture; (**b**) etoposide treated cells or (**c**) PARPi treated cells. Unstained cells and freeze-killed cells were used as negative and positive staining controls respectively (prepared from untreated 3D co-cultured cells). Data are presented as mean ± SD. Significance compared to vehicle control (one way ANOVA with Dunnett’s post-test *p < 0.05; ***p < 0.001).

**Figure 3:**
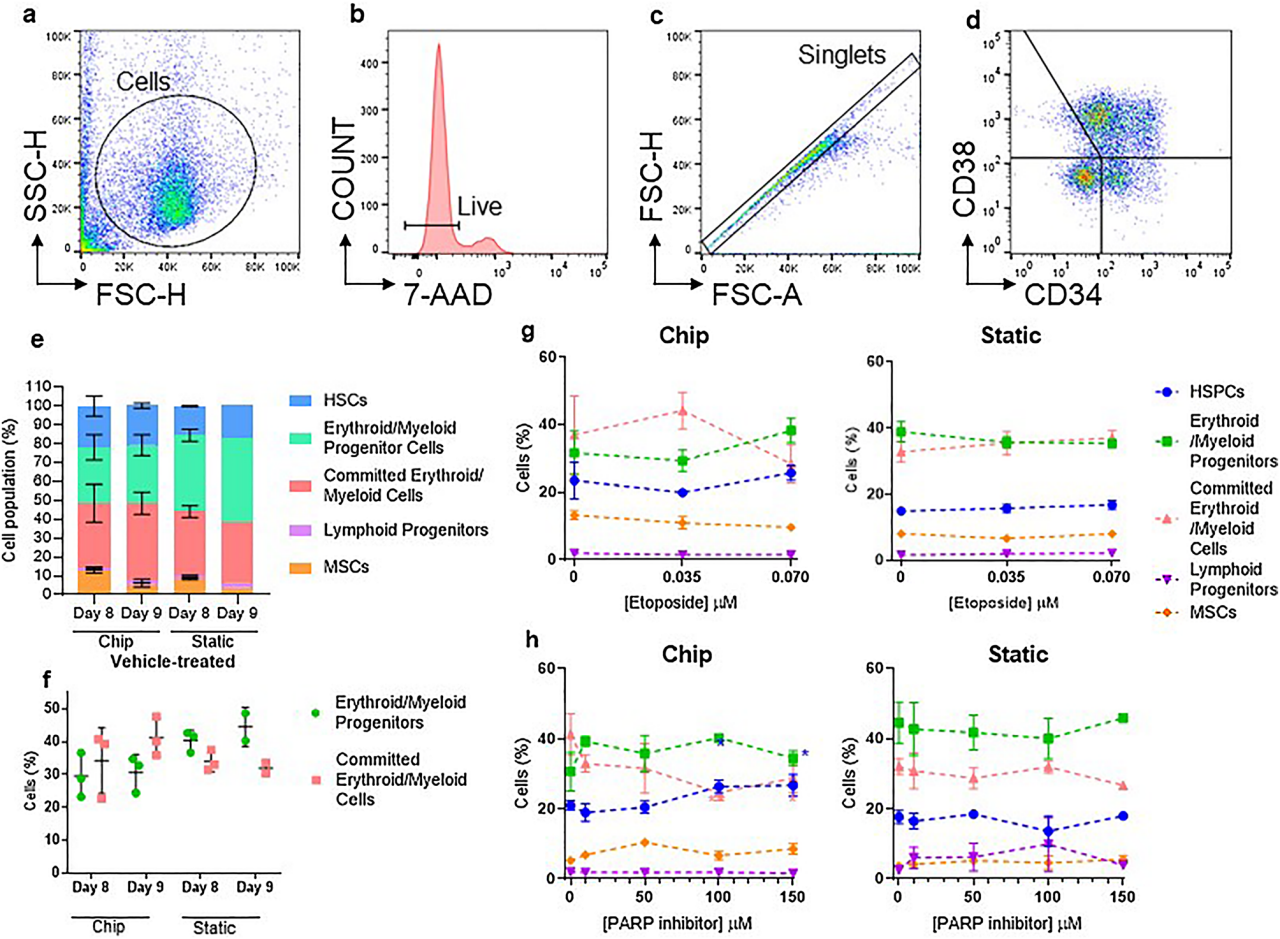
HSPCs within the 3D bone marrow models become differentiated into Erythroid/Myeloid cells, and the chip model drives differentiation further compared to the static. Harvested cells were characterised by flow cytometry using the markers CD34, CD38, CD73 and CD10. Example gating strategy (**a**–**d**) representative of all samples (doublet exclusion only performed on prednisolone-treated static model): CD34/CD38 gate on vehicle-treated controls, determined by FMO controls after 7-AAD viability dead cell exclusion; the percentages of HSPCs (CD34^+^CD38^−^; blue); Erythroid/Myeloid Progenitors (CD34^+^CD38^+^; green), Committed Erythroid/Myeloid cells (CD34^−^CD38^−^ and CD34^−^CD38^+^; red); Lymphoid Progenitors (CD34^+^CD38^−^CD10^+^ and CD34^+^CD38^+^CD10^+^; purple) and MSCs (CD34^−^CD73^+^; orange) following (**e**–**f**) vehicle-treatment (days 8 and 9), (**g**) chip and static models, (**f**) in the chip and static models, following treatment with (**g**) etoposide or (**h**) PARPi. Vehicle-treated conditions: chip days 8 and 9, static day 8 (*n* = 3), static day 9 (*n* = 2). Treated conditions (*n* = 3), except *n* = 2 for 0 μM and 150 μM PARPi treatment in static. Error bars represent the mean ± SD.

To confirm the increases in MN observed in the 3D models were true genotoxic responses to the compounds as opposed to artefacts of high cytotoxicity, cell viability was measured by flow cytometry using the viability stain 7-AAD to give the percentage of viable cells (Fig. 2; 7-AAD measurement by flow cytometry shown in Fig. 3). Freeze-killed cells were used as a staining control for the 7-AAD stain, and a clear reduction (66.9%) in cell viability was observed in these cells. High cellular viability was maintained in untreated cells in both chip and static models at 8 days post HSPC-MSC co-culture initiation (the time of MN scoring for etoposide) of culture (Fig. 2a). The viability is comparable in both 3D chip and static models, with 96.5% ± 0.7% live cells in the chip model and 96.9% ± 0.6% live cells in the static model. This demonstrates that the extended 3D cell co-culture does not have a significant effect on viability. Following treatment, cell viability, expressed as relative viable cells (%), was calculated from the percentage of dead cells indicated by 7-AAD^+^ staining in treated samples compared to vehicle controls. While a statistically significant dose-dependent decrease in relative viable cells was observed in the chip model following PARPi treatment at all doses tested, and a statistically significant decrease in relative viable cells was observed in both models following etoposide treatment at 70 µM (Fig. 2b, c), viability was maintained at above 94.3% in the etoposide-treated static model, and 80.1% in the PARPi-treated chip model.

### The 3D bone marrow models drive differentiation of HSPCs into erythroid and myeloid cells, and the chip model drives the differentiation further than the static model

To explore the cell populations present at the time of MN scoring (8–9 days of HSPCs in co-culture with MSCs, dependent on treatment and harvest schedules), cells were stained for CD34, CD38, CD73, and CD10, as well as 7-AAD for viability and analysed by flow cytometry (Fig. 3). The gating strategy defined cells broadly into HSPCs (CD34^+^CD38^−^), erythroid/myeloid progenitor cells (CD34^+^CD38^+^) and committed erythroid/myeloid cells (CD34^−^CD38^−^ and CD34^−^CD38^+^) (Fig. 3a–d). Lymphoid progenitor cells were also defined by CD10 staining (CD34^+^CD38^−^CD10^+^ and CD34^−^CD38^−^CD10^+^).

Interestingly, in the vehicle-treated controls, there was a trend for a greater percentage of erythroid/myeloid progenitors in the static model compared to the chip model on day 8, although this was not statistically significant (40.3% c.f. 29.6% respectively). This trend was also apparent on day 9 (42.0% c.f. 30.63%) along with a trend for a greater percentage of committed erythroid/myeloid cells in the chip compared to the static model (41.3% c.f. 32.0%). However, these should be viewed with caution as there are only n = 2 for the static model on day 9 (Fig. 3e, f). A very low percentage of lymphoid progenitors and MSCs were found in both models. Following treatment, an apparent decrease in committed erythroid/myeloid cells was observed in the chip model in response to etoposide (23.2% reduction from 0 to 0.070 μM) with a corresponding increase in HSPCs and progenitors, although this was not statistically significant (Fig. 3g). Additionally, a decrease in committed erythroid/myeloid cells was observed in the chip model following treatment with PARPi (30.3% reduction from 0 to 150 μM; Fig. 3h), with a corresponding increase in HSPCs, which was statistically significant for both cell types at 100 µM and 150 µM. This effect was not observed in the static model.

### 3D bone marrow micronucleus trends are comparable to in vivo

**Figure 4:**
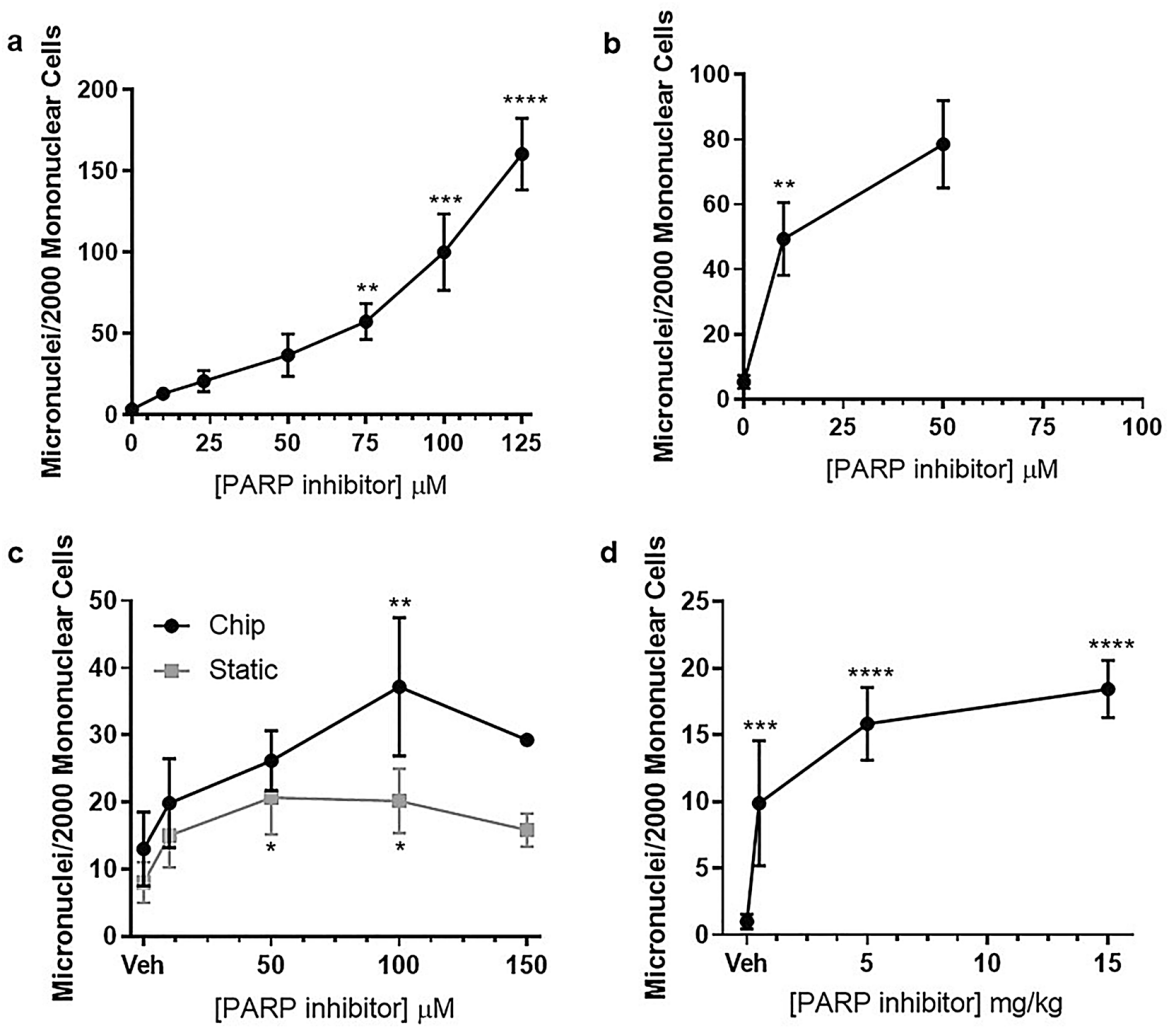
A dose-dependent increase and plateau in micronucleus (MN) frequency is observed in 3D bone marrow models and in vivo, in response to PARPi treatment. MN levels following PARPi treatment were compared between 2D L5178Y (**a**), 2D BM cells (**b**), 3D (chip and static) (**c**) and in vivo (**d**). Data were recorded as MN/2000 mononuclear. 2D and 3D conditions: *n* = 3 (*n* = 2 for 150 µM PAPRi in chip and 50 µM in 2D BM cells); In vivo groups: *n* = 5. Data are presented as mean ± SD. Significance compared to vehicle control (one-way ANOVA with Dunnett’s post-test (*p < 0.05; **p < 0.01; ***p < 0.001; ****p < 0.0001).

To compare trends of MN levels with dose between the different in vitro models and in vivo, the MN data for the PARPi were re-plotted as a line graph (Fig. 4). These plots show that in the L5178Y assay there is a dose-dependent increase (Fig. 4a), in the 2D BM assay, there is an increase in MN from 0 to 10 µM which tapers off at 10–50 µM (Fig. 4b), and in the 3D models there was an increase in MN from 0 to 50 μM followed by a plateau/reduction at 50–150 μM (Fig. 4c). The profile observed in the 2D BM assay resembles the initial phase of the in vivo profile, but the profile in the 3D models is more comparable to that observed in vivo (Fig. 4d).

### 3D bone marrow models detect MN after treatment with prednisolone

**Figure 5:**
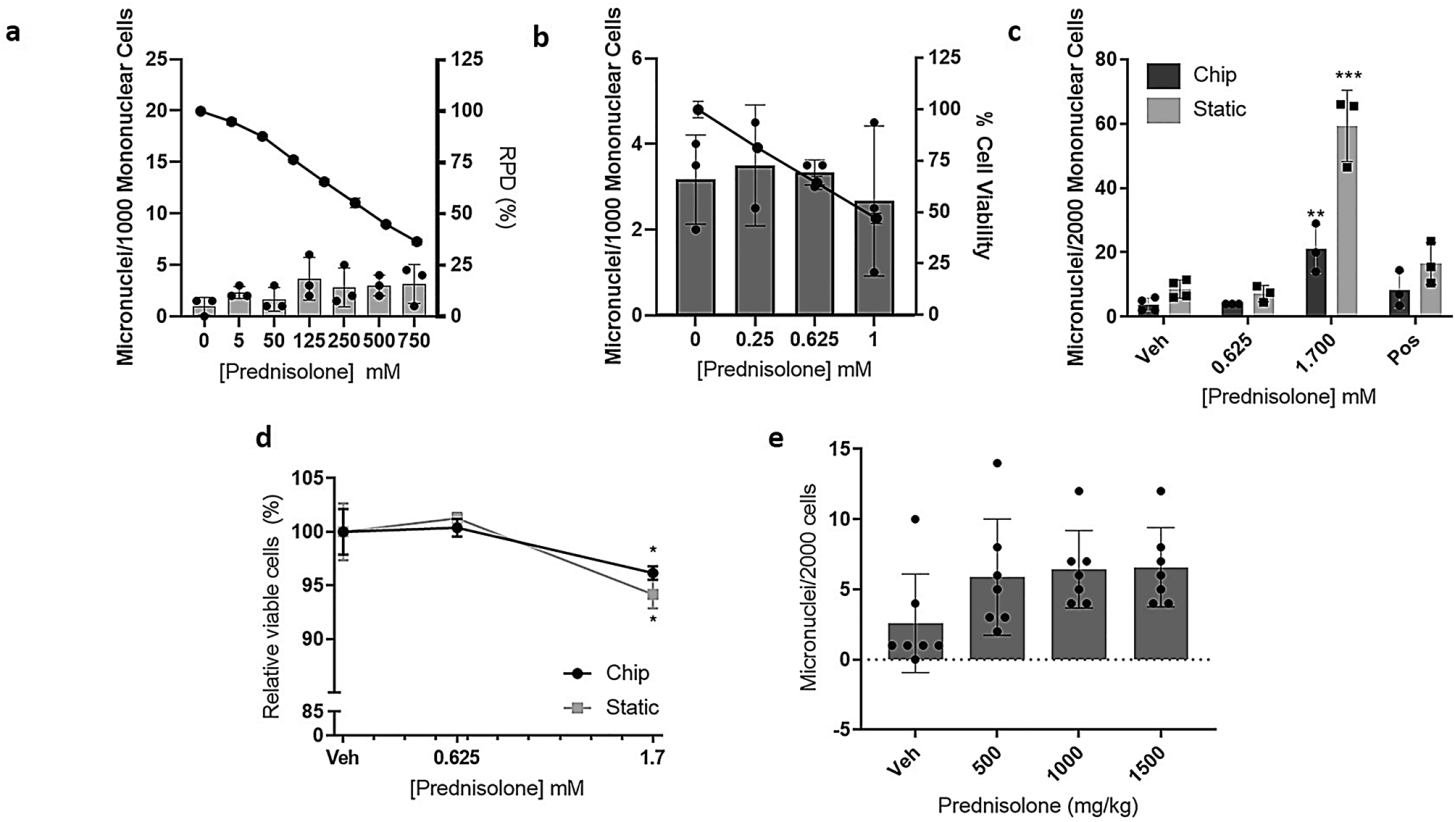
3D bone marrow (BM) models detect micronuclei (MN) after treatment with prednisolone. (**a**) MN and cytotoxicity were measured in the in vitro L5178Y MN assay. Data are presented as MN/1000 mononuclear cells (bars) and lie within the historical vehicle-treated control for the laboratory (indicated by the dashed lines; 2.85 ± 2.26). Cytotoxicity is presented as the RPD (line). (**b**) MN and cytotoxicity were measured in the 2D BM assay. Data are presented as MN/1000 mononuclear cells (bars) and cytotoxicity as percentage cell viability (line). (**c**) MN following treatment of 3D BM models with 0–1.7 mM prednisolone. Data are presented as MN/2000 mononuclear cells. (**d**) Cytotoxicity of cells from the 3D BM models was measured by flow cytometry 7-AAD^+^ staining, identifying dead cells. Data are presented as the percentage of relative live cells. (**e**) Previously acquired in vivo data (MN/2000 cells following treatment of rats with 0–1500 mg/kg prednisolone). 2D and 3D conditions: *n* = 3 (*n* = 2 for 0.25 µM prednisolone in 2D BM cells). In vivo groups: *n* = 7. Data (except (**e**)) are presented as mean ± SD. Significance compared to vehicle control (one-way ANOVA with Dunnett’s post-test (**p < 0.01; ***p < 0.001; ****p < 0.0001).

To investigate whether the 3D BM models could detect increases in MN following treatment with drugs that have pharmacological effects on the BM resulting in an increase in MN in vivo only, the chip and static 3D models, as well as the 2D L5178Y and 2D BM cells, were treated with prednisolone. Prednisolone is positive in vivo due to pharmacological effects on the BM (Fig. 5e), but negative in vitro. The results confirm that prednisolone is negative in the 2D in vitro assays: L5178Y cells (Fig. 5a) and 2D BM MN cells (Fig. 5b). In both chip and static models, prednisolone (1.7 mM) induced a statistically significant increase in MN (5.60 fold in the chip and 6.88 fold increase in the static, *p* < 0.01 and p < 0.001 respectively) (Fig. 5c). Cytotoxicity, measured using 7-AAD staining by flow cytometry, indicated that < 50% cytotoxicity was induced by prednisolone in both models (no cytotoxicity at 0.625 mM, 4.97% and 7.24% cytotoxicity was observed at 1.7 mM treatment in the chip and static models, respectively) (Fig. 5d). It is noteworthy that the increase in MN in the chip and static models is greater than that observed in vivo (6.9× and 5.6× c.f. 2.5× respectively).

## Discussion

There is a need for a more physiologically-relevant in vitro model to enable earlier detection of late-stage in vivo pharmacological MN positives that do not indicate potential genotoxicity but prevent progression of these non-genotoxic drugs into the clinic.

In the current study we compared a 3D BM chip model (in a microfluidic system) and a 3D static model (in static culture) for MN detection. The results show that both models can detect MN following treatment with etoposide or a PARPi, both of which induce DNA damage, but by different mechanisms (etoposide inhibits Topoisomerase II^19^ while the PARPi increases DNA damage and error-prone repair in HR-deficient cells by NHEJ^20^ as detailed in the introduction). This indicates that our 3D BM models can detect DNA damage caused by different mechanisms.

In vivo, the PARPi induces an increase in MN followed by a plateau. This is in contrast to the 2D L5178Y cells where there is a dose-dependent increase in MN for all doses tested. Interestingly, the profile of MN induction in the 3D BM models was more similar to in vivo than 2D L5178Y cells, suggesting that our human 3D BM models recapitulate the in vivo scenario and the cells have a response to genotoxins that is more comparable to that observed in vivo. In support, the reconstituted skin models have been suggested to be more physiologically relevant compared to current in vitro assays for dermally-applied chemicals^22^ and nanoparticles^23^. It is worth noting that the profile in the 2D BM cell model is somewhat comparable to the initial part of the MN induction profile in vivo, however, a plateau was not observed in the 2D BM cell assay. While this may require testing higher doses, since the plateau in the 3D models was at doses > 50 µM, 50% cytotoxicity was observed at the 50 µM dose in the 2D BM assay, and as such higher doses cannot be tested for MN induction in this assay.

Having determined the ability of the 3D models to detect MN following exposure to known genotoxins, we tested whether these models could detect MN following exposure to prednisolone, which is negative in vitro (2D L5178Y) but positive in vivo. The results show an increase in MN in both 3D models following treatment, suggesting these 3D models recapitulate the in vivo environment sufficiently to enable detection of compounds only positive in vivo. Importantly, prednisolone was also negative in the 2D BM model, suggesting that it is the 3D microenvironment that is important here, rather than cell type. The in vivo positive is believed to be due to a secondary effect of the pharmacological mechanisms on the IE cells^5,6,24^, rather than due to cytotoxic artefacts or true genotoxicity; steroids are considered not to be genotoxic^4^. Since prednisolone was positive in 3D but not in the 2D BM assay, these data, albeit from one compound, suggest that these 3D BM models sufficiently recapitulate the in vivo BM environment to allow detection of compounds that are in vivo pharmacological positives. Further testing with other compounds only positive in vivo is required to confirm this. Indeed, both 3D models drove differentiation of HSPCs down the erythroid/myeloid lineage, while still maintaining a HSPC pool, suggesting that co-culture of HSPCs with MSCs on a hard, porous scaffold in cytokine-containing media provides a 3D microenvironment sufficient for stem cell maintenance with concurrent differentiation, which is in agreement with the study by Sieber et al.^18^. Interestingly, in vehicle-treated conditions, our data suggest that the fluidic chip model contained a higher percentage of committed erythroid/myeloid cells compared to the static model, which maintained a greater percentage of erythroid/myeloid progenitor cells. It is considered that the addition of microfluidics increases the physiological relevance of 3D culture; for example, Bruce et al.^25^ showed that a 3D microfluidic model created a more accurate niche compared to a 2D or 3D static model. Stem cell culture and differentiation require precise control of multiple cues in the cell culture microenvironment, and in microfluidic devices the microenvironment, including flow rate and soluble factors, can be precisely controlled to direct stem cell differentiation^13^. A number of studies have reported stem cell differentiation in microfluidic devices, including neuronal stem cells^26,27^ and endoderm-directed differentiation of mouse embryonic stem cells^28^. It is possible that the addition of flow in our BM chip model further recapitulates the in vivo microenvironment improving stem cell differentiation, however, these data are only n = 2 and further investigation is required to conclude this. Very few lymphocyte-lineage cells were detected, however this is not surprising since it has been shown that IL-7, among other cytokines, is critical for lymphocyte development^29^, and this is not present in the cytokine cocktail used in the current study, demonstrating the specificity of the erythroid/myeloid cytokine-inducing media used. Furthermore, the differentiation indicates that cells are proliferating in the 3D models, which is critical for a MN assay, as cell division is required for MN to form.

Interestingly, the magnitude of response was similar in both 3D models for each compound tested, suggesting that for the compounds tested in the current study, the addition of microfluidics does not alter the response of these cells to DNA damaging agents, despite our results indicating increased differentiation in the chip, most likely because these are short-term effects. Given the current high cost and complexity of these 3D microfluidic studies, utilising a 3D static, rather than fluidic model for genetic toxicity testing would be advantageous. However, it should be noted that a limited selection of genotoxic compounds has been tested in the current study, and other compounds with different mechanisms of action should be assessed to confirm whether or not fluidics is required for MN assessment.

It is also interesting to note that the magnitude of MN response detected in the 3D models following exposure to etoposide and PARPi was lower compared to both 2D assays for equivalent doses. One possible reason is that the BM cells may be less susceptible to DNA damage in their 3D microenvironment compared to the L5178Y cells and the BM cells in 2D culture. In support, it has previously been shown that co-culture of leukemic cell lines with BM MSCs in 3D provided chemoprotection compared to co-culture in 2D or in suspension^30^ and leukemic cells showed decreased sensitivity to chemotherapeutic agents in 3D compared to 2D^25^. Regarding the higher MN levels in L5178Y cells, these cells have a dysfunctional p53^31^, whereas it is expected that the HPSCs used in the current study would be p53 competent, since it has been reported that p53 protects the genetic integrity of HSPCs and cell division of HSPCs promotes the repair of double strand breaks by HR^32^. It might therefore be expected that damaged L5178Y cells would survive rather than undergo cell cycle arrest allowing for DNA repair or apoptosis, while HSPCs would undergo repair, or apoptosis. Alternatively, there could be altered toxicodynamics in 3D, which would influence the MN induction following treatment. For example, the presence of the scaffold and multiple cell types may alter the distribution and exposure of cells to the drug relative to a 2D suspension cell culture.

Cytotoxicity was also measured via 7-AAD staining following treatment in the 3D models and importantly did not reach 50%, showing these models can detect genotoxicity in the absence of excessive cytotoxicity. Freeze-killed cells, used as the 7-AAD staining control resulted in significant cell death (66.9% 7-AAD + cells), demonstrating that this was a reliable measure of cytotoxicity. This not withstanding, 33.1% cells were 7-AAD- in the freeze-killed control, indicating live cells, which is likely due to sub-optimal freezing time. However, viability was calculated for each treatment compared to the vehicle control and clear quantifiable changes in cytotoxicity were still observed. The data also suggest that there is less cytotoxicity following exposure to equivalent doses in the 3D BM cells compared to the 2D BM cells. Culture in 2D is not reflective of the 3D environment in vivo, and studies have reported lower sensitivity of cells cultured in 3D compared to 2D^33^. Moreover, It is also important to note that the chip is formulated from PDMS, which is known to bind small molecules to varying degrees^34,35^. While it is possible that some of the drugs used in the current study could have bound to the PDMS in the chip, resulting in a lower concentration in the media and consequently reduced MN induction and cytotoxicity, the absence of PDMS in the 3D static model and the similar magnitude of response seen in both 3D models suggests limited drug binding to PDMS. This would need to be confirmed by bioanalysis of drug in the media.

An increase in MN was observed following treatment with 1.7 mM prednisolone, with viability > 90% in both 3D models. The 1.7 mM dose is high compared to the dose required to reach 55 ± 5% cytotoxicity in L5178Y cells of 0.75 mM and the 2D BM cells of 1 mM, and the maximum recommended dose for pharmaceuticals for in vitro mammalian cell assays of 1 mM^36^. However, the OECD guideline states the highest dose should aim to achieve 55 ± 5% cytotoxicity^21^ and cytotoxicity is below this in our study. In support, a higher concentration than was required in 2D was used in the 3D skin model for nanoparticle testing^23^, thus it is likely that the 3D architecture plays a role in the requirement for higher doses in 3D. Furthermore, since the 3D models are more like in vivo than in vitro, the maximum dose and 55 ± 5% cytotoxicity measure for in vitro testing may not be relevant and instead it might be more appropriate to follow the guidelines for the in vivo MN test. The maximum dose and measure of cytotoxicity used in 3D models is an important consideration when thinking about the use of these models in routine genotoxicity testing.

In conclusion we have successfully developed a static and microfluidic 3D primary human BM model and have demonstrated their ability to detect MN following exposure to genotoxic compounds with a profile more similar to in vivo in the rat, highlighting the similarity between the 3D models and in vivo. Importantly, these models can detect MN that are a consequence of pharmacological effects in the BM, which can currently only be detected in vivo, therefore these 3D models could bridge the gap between in vitro and in vivo testing. With ongoing work to further validate these models, ultimately, they could provide a valuable tool to follow up, and eventually predict, suspected pharmacological mechanisms, which could reduce the number of animal studies required.

## Methods

All chemicals and reagents were purchased from Sigma-Aldrich (Dorset, UK) unless otherwise stated.

### 2D cell culture

Mouse lymphoma cells L5178Y Tk^+/−^, clone 3.7.2C, were obtained from Dr J. Cole (MRC Cell Mutation Unit, University of Sussex, Brighton, UK), originally supplied by Dr Don Clive in 1978. The L5178Y cell line was used in the in vitro MN assay. Cells were cultured in RPMI 1640 medium (Dutch Modification), supplemented with 10% heat inactivated donor horse serum (Gibco™, Thermo Fisher Scientific), 2 mmol/l L-glutamine, 2 mmol/L sodium pyruvate, 1% pluronic F68 (Gibco™), 200 IU/mL penicillin and 200 μg/mL streptomycin (termed R10 from here on) and maintained at 37 °C in a humidified environment with 5% CO_2_ in air.

### 2D in vitro MN assay

Cells were seeded at 0.5 × 10^5^ cells/mL in 10 mL R10 medium in T25 flasks and cultured for 24 h following which cells were treated with the compounds or appropriate positive and solvent control as detailed in Table 1 (n = 3 biological replicates) for 24 h. At the end of treatment cells were collected by centrifugation (200×*g*, 5 min), washed once with fresh medium, resuspended at 2 × 10^5^cells/mL in 10 mL fresh medium and incubated for a 24 h recovery period. Following recovery, cells were counted, and the concentration adjusted to 1 × 10^5^cells/mL in 3 mL of R10 media containing 2% pluronic and cytospun onto slides. Cells were fixed in 100% methanol and stained with acridine orange. The frequency of MN in mononuclear cells was scored blind in 2000 cells per sample. Cytotoxicity was assessed using relative population doubling (RPD), which was calculated using the following equations:

$$\begin{aligned} Population\,doubling & = \left[ {\frac{Post\,treatment\,cell\,number}{{Initial\,cell\,number}}} \right] \div log2 \\ RPD & = \left( {\frac{Number\,of\,population\,doublings\,in\,treated\,cultures}{{Number\,of\,population\,doublings\,in\,control\,cultures}}} \right) \times 100 \\ \end{aligned}$$

**Table 1:** Compounds used in the 2D and 3D assays.

Compound	Concentration (µM)	Vehicle	Vehicle concentration (%)	Assay	Treatment schedule
Etoposide (Sigma-Aldrich)	0–0.070	Dimethyl sulfoxide; DMSO (Sigma-Aldrich)	0–0.000396	3D BM-on-a-chip; 3D BM static culture	24 h treatment (1 dose)
	0–0.065	DMSO	1	2D BM culture; 2D L5178Y	24 h treatment (1 dose) + 24 h recovery
AZ12785452; an undeveloped PARPi (AstraZeneca)	0–150	DMSO	0–0.123	3D BM-on-a-chip; 3D BM static culture	48 h treatment^┼^ (2 doses, 1 at 0 h and a second at 24 h)
	0–125	DMSO	1	2D BM culture; 2D L5178Y	24 h treatment (1 dose) + 24 h recovery
Prednisolone (Sigma-Aldrich)	0–1700	DMSO	0–0.86*	3D BM-on-a-chip; 3D BM static culture	24 h treatment (1 dose)
	0–1000	DMSO	1	2D BM culture; 2D L5178Y	24 h treatment (1 dose) + 24 h recovery
Mitomycin C; MMC (Sigma-Aldrich)	0.04	Sterile H_2_0	1	2D L5178Y (positive control)	24 h treatment (1 dose) + 24 h recovery

^┼^Dosing regimen was selected to align with that used in the in vivo study.

*The highest DMSO concentration used in the 3D assays did not negatively affect cell viability as tested by 7-AAD^+^ staining in flow cytometry.

### 2D BM cell in vitro assay

CD34^+^ Haematopoietic stem and progenitor cells (HSPCs; Lonza, Cambridge, UK) were used for the 2D BM in vitro MN assay. Cells were seeded at 10,000 cells/well in a 12 well plate in burst-forming unit-erythroid (BFU-E) media and cultured for 3 days. Following this, cells were collected by centrifugation (300×*g*, 15 min) and seeded at 32,000 cells/well in 24 well plates. Cells were treated with the compounds or appropriate positive and solvent control as detailed in Table 1 (n = 3 biological replicates) for 24 h. At the end of treatment, cells were collected by centrifugation (300×*g*, 5 min), washed three times with fresh Iscove's Modified Dulbecco's Media (IMDM), re-plated in 24 well plates in 500 µl fresh medium and incubated for a 24 h recovery period. Following recovery, 100 µl cells were transferred to a transparent-bottomed white-walled 96 well plate for ATP analysis using the CellTiter Glo assay (Promega, Southampton, UK) following manufacturer’s instructions with slight modifications. Briefly, 10 µl of ATP substrate was added in 100 µl of media in a 96 well plate and the luminescence was measured using an Envision (Perkin Elmer, Beaconsfield, UK). Luminescent signal, which linearly reflects the number of cells, was used to calculate the percentage cell viability. The remaining 400 µl of cells were assessed for MN as described for L5178Y cells above (2D in vitro MN assay) with the exception that cytotoxicity was calculated by ATP analysis as described above.

### 3D bone marrow model culture

**Figure 6:**
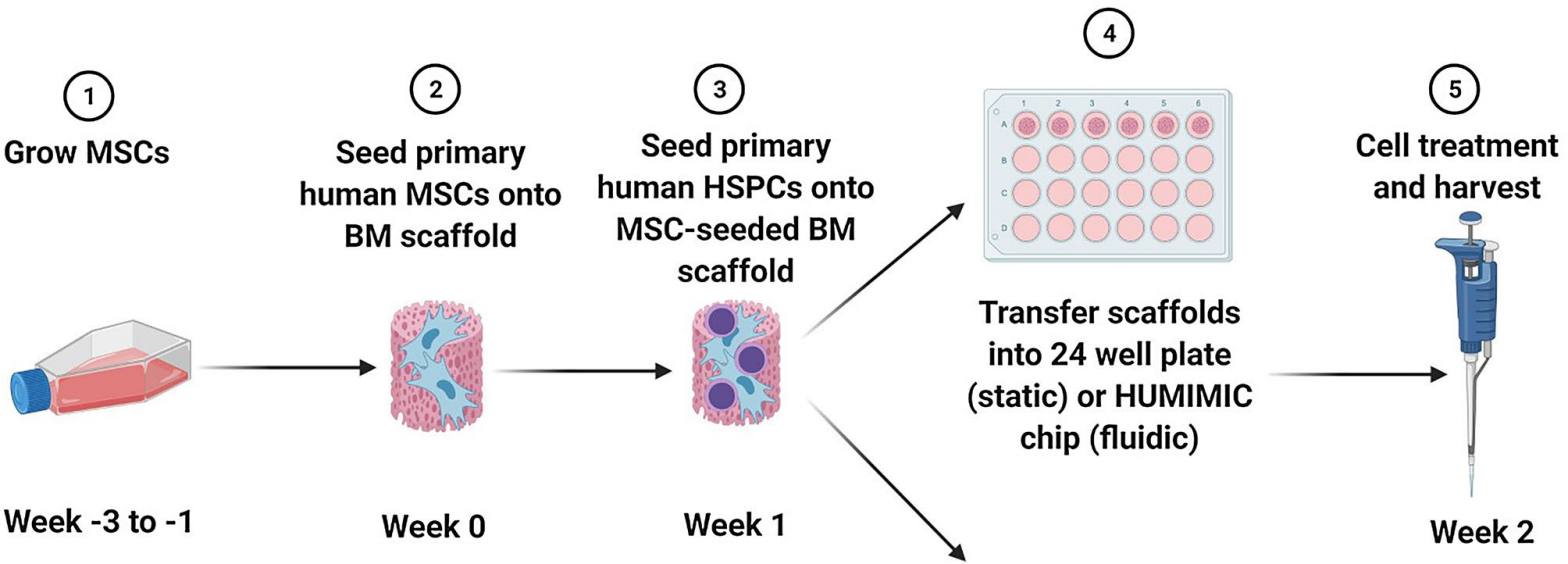
Schematic representation of 3D model study schedule. Created with BioRender.com.

3D BM model studies were carried out as outlined in Fig. 6. Human Mesenchymal Stem Cells (MSCs; Lonza) were cultured in Dulbecco’s Modified Eagle Medium (DMEM high glucose), supplemented with 5% (v/v) human platelet lysate (hPL; STEMCELL technologies, Cambridge, UK), 200 IU/ml penicillin, 200 μg/mL streptomycin and 1% (v/v) Glutamax (termed D10 from here on), and maintained for < 5 passages post-thawing. A single MSC donor was used per experiment. MSCs (0.5 × 10^6^ cells) were seeded onto hydroxyapatite‐coated zirconium oxide‐based Sponceram® 3D ceramic scaffolds (Zellwerk GmbH, Germany) with a diameter and height of 5.8 mm in a 96 well ultra-low-attachment plate (ULA; Corning Biosciences, New York, USA) in D10 excluding hPL. After 5–16 h, media and MSC-coated ceramics were transferred to a 24 well ULA plate (Corning Biosciences) and media added to a total volume of 1.5 mL. MSCs were cultured in the ceramics for 1 week post-seeding with 100% media changes every 48 h.

After 1 week, ceramics were transferred to a 96 ULA plate, the medium removed, and CD34^+^ HSPCs cells (Lonza) were seeded onto the MSC-coated ceramics at 1 × 10^4^/200 μL per ceramic in StemSpan™ Serum-Free Expansion Medium (SFEM II; STEMCELL Technologies) supplemented with the following cytokines and final concentrations: 0.02 μg/mL EPO, 0.05 μg/mL SCF, 0.1 μg/mL Flt-3-L, 0.1 μg/mL TPO and 0.001 μg/mL G-CSF^37^ (all from PeproTech, London, UK). A single HSPC donor was used per experiment. HSPCs were incubated in the scaffold for 5–16 h following which 3D cultures were established. To set up the chip (3D BM-on-a-chip) model, MSC-HSPC coated ceramics were transferred into one compartment per circuit in the HUMIMIC Chip2 (TissUse GmbH, Berlin, Germany), while the other compartment served as a medium reservoir and 400 μL SFEM II medium was added to each compartment of each circuit. The flow rate was set to 5 µL/min^18^ and the flow of recirculating media was directed through the medium reservoir first. To set up the static (3D BM static culture) model, MSC-HSPC coated ceramics were transferred to 24 well ULA plates and 1.5 mL cytokine-supplemented SFEM II medium added. Cultures were maintained for 7 days with 50% media changes every 48 h prior to dosing with etoposide, the PARPi or prednisolone with the doses and schedules detailed in Table 1, n = 3. The donor and demographic information for the cells used in the study is detailed in Table 2. The authors refer the reader to the publication by Sieber et al. for images of the scaffold and chip used in the current study^18^.

**Table 2:** Cell donor information.

Experiment	Compound	Study design	MSC				HSC			
			Donor (Lot #)	Sex	Age	Race	Donor (Lot #)	Sex	Age	Race
MN assay—3D Chip and static	Prednisolone	MN assessment Cell viability (flow)	0000602009	M	23	B	0000573127	M	26	A
MN assay—3D Chip and static (Etoposide Exp 4)	Etoposide	MN assessment Cell viability (flow) Cell composition assessment (flow)	0000636886	M	44	B	0000349900	F	20	B
MN assay—3D Chip and static (PARPi Exp 3)	PARPi	MN assessment Cell viability (flow) Cell composition assessment (flow)								

*F* female, *M* male, *A* Asian, *B* Black.

Following treatment, cells were collected from the scaffold by flushing the ceramic with 400 μL phosphate-buffered saline with EDTA and 3% Bovine Serum Albumin (BSA) (PBE) and an additional 1.4 mL PBE was added before incubation at 37 °C for 15 min. The PBE solution and the ceramic were centrifuged for 5 min at 300×*g*. Supernatant was discarded, and the pelleted cells were combined, divided into two samples, one resuspended into a total volume of 1 mL PBE (for flow cytometry) and the other into 800 μL IMDM (for MN assessment). Cells were assessed for MN as described for L5178Y cells above (2D in vitro MN assay) with the modification that 4000 cells were scored.

### Flow cytometry

To assess the cellular composition and viability in the scaffolds, cells were stained with the DNA-intercalating viability dye 7-AAD, and the antibodies CD34-ApCCy7, CD38-APC, CD73-FITC and CD10-PE (all from BioLegend, London, UK). Briefly, cells were washed once (300×*g*, 5 min, 4 °C), resuspended in PBS-0.5% BSA and placed on ice. Antibody cocktails (4 colour and fluorescence minus one (FMO)) were prepared in the dark, 250 μL cell samples were transferred to a 96 well round bottom ULA plate (Corning) and stained with antibody cocktails at 4 °C for 30 min in the dark. Plates were washed twice (300×*g*, 5 min, 4 °C) and pellets resuspended in 250 μL fresh PBS-0.5% BSA before acquisition on the Guava easyCyte 8HT flow cytometer (Millipore, Burlington, MA, USA). Guava single stained beads were used as antibody compensation controls following manufacturer’s instructions (AbC total Antibody Compensation Bead Kit; Thermo Fischer Scientific, Waltham, MA, USA). Freeze-killed 3D model-harvested cells were used for the 7-AAD compensation control (stain dead cells), vehicle-treated cells were used for the unstained control. Data were analysed using FlowJo 10 (FlowJo LLC, Ashland, OR, USA).

### Animal data

All animals were treated in accordance with approved UK Home Office licence requirements. All experimental protocols were approved by AstraZeneca. All methods were carried out in accordance with current Organisation for Economic Cooperation and Development (OECD) guidelines, except that concurrent positive control groups were not included in the standard protocol in use at AstraZeneca.

The animal data for prednisolone used for comparison to in vitro data in the current study were previously obtained in accordance with ARRIVE guidelines as described in Hayes et al.^3^.

The animal data for AZ12785452 used for comparison to in vitro data in the current study were previously obtained in accordance with ARRIVE guidelines using the study design outlined in Hayes et al.^3^ except that animals were killed by administration of isoflurane followed by severance of major blood vessels.

### Statistics

Data are depicted as mean ± standard deviation (SD). Statistical analysis was performed using GraphPad Prism software (version 7.04). Statistically significant increases in MN and decreases in cytotoxicity were determined using a one-way analysis of variance (ANOVA) with Dunnett’s post-test by comparing compound-treated conditions to vehicle controls. Values of *p* < 0.05 were considered statistically significant.
